# Gyejibongnyeong-Hwan (Gui Zhi Fu Ling Wan) Ameliorates Human Uterine Myomas *via* Apoptosis

**DOI:** 10.3389/fphar.2019.01105

**Published:** 2019-09-25

**Authors:** So Min Lee, Eun Som Choi, Eunyoung Ha, Kon Young Ji, So Jin Shin, Jeeyoun Jung

**Affiliations:** ^1^Clinical Medicine Division, Korea Institute of Oriental Medicine, Daejeon, South Korea; ^2^Department of Obstetrics and Gynecology, Institute for Cancer Research, Keimyung University School of Medicine, Daegu, South Korea; ^3^Department of Biochemistry, Institute for Cancer Research, Keimyung University School of Medicine, Daegu, South Korea; ^4^Herbal Medicine Research Division, Korea Institute of Oriental Medicine, Daejeon, South Korea

**Keywords:** uterine leiomyoma cell, Gyejibongnyeong-hwan, apoptosis, bax, Bcl-2

## Abstract

Uterine leiomyomas are the most common benign neoplasms in women of reproductive age. However, non-surgical treatments for uterine myomas have not been fully evaluated. In Korea and China, Gyejibongnyeong-hwan (GBH) is widely used to treat gynecological diseases. Thus, we investigated the effects of GBH in human uterine myoma cells (hUtMCs). The hUtMCs were collected from patients undergoing curative surgery. Cell viability was analyzed *via* 3-(4,5-dimethylthiazol-2-yl)-2,5-diphenyltetrazolium bromide (MTT) assays. The expression levels of p53, Bax, Bcl-2, cleaved-caspase-3, and caspase-9 were determined by Western blotting. Apoptosis and ROS levels were evaluated by fluorescence microscopy. First, we determined the adequate concentration that did not affect normal cells, and then investigated the time-dependent anti-neoplastic effect of GBH to decide the appropriate treatment time under a non-toxic concentration. Cell viability and number were significantly reduced by GBH at 48 h in a dose-dependent manner (0–200 µg/ml). The ratio of Bax to Bcl2 and expression of p53, cleaved-caspase-3, and caspase-9 increased, representing GBH induced apoptosis in uterine leiomyomas. In addition, preliminary tests using pan-caspase inhibitor/p53 inhibitor with GBH rescued the GBH-mediated apoptotic effect. Furthermore, GBH significantly increased the mitochondrial ROS concentration, and preliminary test showed that mitochondria ROS scavenger reduced the percentages of early apoptosis cell. These results suggest that GBH may induce apoptosis of leiomyomas and demonstrated that GBH can be a potential therapeutic and/or preventive agent for uterine leiomyomas.

## Introduction

Uterine leiomyomas (fibroids or myomas) are very common benign gynecological tumors affecting women of reproductive age (Stewart, 2001; Laganà et al., 2017). Approximately 25% of women have clinically significant lesions (Tinelli and Malvasi, 2015), and pathological examinations of surgical specimens suggest that the prevalence of myomas is as high as 77% (Cramer and Patel, 1990).

Myomas can cause abnormal uterine bleeding, pelvic pressure and pain, and reproductive dysfunctions, and they are a major indication for hysterectomy, accounting for over 200,000 cases annually in the United States (Walker and Stewart, 2005). The most common treatment option for leiomyomas is surgery. Surgical options include myomectomy or hysterectomy and, more recently, uterine artery embolization and focused ultrasound surgery. However, these procedures are unacceptable to many women who wish to retain their uterus (Laganà et al., 2017), and pharmacological alternatives are limited.

Gonadotropin-releasing hormone agonists have been used for short-term therapy to reduce myomas, but they cause substantial side effects and adverse reactions, such as loss of bone mass and hot flashes (Kouides et al., 2009; Kriplani et al., 2009). Progesterone-containing agents (norethisterone), levonorgestrel-releasing intrauterine devices, and other hormonal therapies are used to reduce symptoms attributable to myomas; however, each of these strategies has limitations (Irvine et al., 1998; Reid and Virtanen-Kari, 2005; Sankaran and Manyonda, 2008). Only ulipristal acetate (UPA) has been approved in Europe for preoperative fibroid treatment since 2012 (Pohl et al., 2013; Donnez et al., 2015). However, it causes several side effects, such as headache, nausea, and abdominal pain (Glasier et al., 2010).

To develop better therapeutics against uterine fibroids, we focused on the traditional Korean and Chinese medicine, Gyejibongnyeong-hwan (GBH). GBH was first reported in *Shanghan Lun* edited by Zhang Zhongjing of the Han Dynasty and has been considered as an effective agent for treating gynecological diseases, such as period pain, dysmenorrhea, and emmeniopathy (Choi et al., 2008; Liu et al., 2013). In addition, a previous study has reported that GBH significantly improves uterine myoma volumes and decreases abnormally increased hormone levels (Chen et al., 2014). However, the mechanisms underlying the effects of GBH on uterine myomas are yet to be determined.

Apoptosis plays an important role in the pathogenesis of tumor cells (Elmore, 2007). Under normal conditions, cell division and cell death are tightly regulated (Wong, 2011). When cells are no longer needed, apoptosis is initiated, marked by changes in the morphology, including membrane blebbing, cell shrinkage, chromatin condensation, apoptotic body formation, and DNA damage, *via* a p53-dependent pathway (Nagata, 2000). There are two basic apoptotic signaling pathways: pathways mediated by mitochondria (intrinsic) and pathways mediated by death receptors (extrinsic). The intrinsic apoptotic pathway is triggered by different intracellular stimuli and is characterized by Bax translocation; cytochrome c release from the mitochondria; and activation of the initiator caspase-9, the executioner caspases-3 and -6, and poly(ADP-ribose) polymerase(PARP) cleavage (Nagata, 2000; Youle and Strasser, 2008).

Therefore, in this study, we evaluated the effects of GBH on primary cultured leiomyoma cells and also investigated the mechanism underlying the effects of GBH in a comparative analysis with UPA, which has pro-apoptotic effects by upregulation of cleaved caspase-3 and downregulation of Bcl-2 expression on leiomyoma cells (Yoshida et al., 2010; Horak et al., 2012). The findings of our study may be useful in developing potent therapeutic agents containing GBH for the treatment of uterine myomas.

## Methods

### Preparation of the GBH Decoction

The GBH decoction was prepared by extracting a mixture of five types of dried medicinal herbs, which included the following: 4 g of *Cinnamomum cassia* Blume, 4 g of *Poria cocos*, 4 g of *Paeonia suffruticosa* Andrews, 4 g of *Paeonia lactiflora* Pallas, and 4 g of *Prunus semen*. All the herbal components were purchased from Omniherb (Daegu, Korea). A voucher specimen (GBH-1) was deposited at the Herbarium of the Korea Institute of Oriental Medicine.

We prepared GBH extract according previous studies (Yi et al., 2002; Jeong et al., 2015; Vakifahmetoglu-Norberg et al., 2017;Lee et al., 2018). Briefly, GBH comprising five herbal medicines was mixed (2 kg; 4 g × 5 herbs × 100-fold) and extracted in a 10-fold mass in distilled water (20 L) at 100°C for 2 h. The aqueous extract was filtered by pressing through a filter (10 μm pore size), and then the solution was evaporated and freeze-dried to give a powder. The amount of GBH powder was 181.48 g (extract yield, 9.88%), which was then stored at −70°C until use.

### Liquid Chromatography-Mass Spectrometry for GBH Analysis

The chemical profile of GBH was examined by liquid chromatography-mass spectrometry (LC-MS). Standard chemicals (paeoniflorin, ferulic acid, cinnamic acid, 18-iso-rhamnetin-3-*O*-rutinoside, albiflorin, coumarin cinnamaldehyde, amygdalin, paeonol, and vanillic acid) were purchased from NPC BioTechnology, Inc. (Daejeon, Korea). The high-performance liquid chromatography (HPLC)-grade solvents were obtained from Honeywell Burdick & Jackson (Muskegon, MI, USA).

For the liquid chromatography-Mass spectrometry/Mass spectrometry (LC-MS/MS) analysis, 1 ml of acetonitrile was added to 10 mg of the freeze-dried GBH powder and sonicated for 30 min at room temperature (23°C). The solution was purified using a 0.2-µm Teflon syringe filter (Thermo Fisher Scientific, Waltham, MA) to eliminate dust and other particles. The sample was then transferred to an LC glass vial, and 10 µL of the sample was injected for each LC-MS/MS analysis.

LC-MS was performed using the Thermo Vanquish ultra high-performance liquid chromatography (UHPLC) (Thermo Fisher Scientific), and the AB SCIEX 3200 QTRAP system (AB Sciex, Concord, Canada). Multiquant (version 3.0.2; AB Sciex, Concord, Canada) was used for data acquisition and analysis.

LC separation was performed using a ZORBAX Eclipse Plus system (2.1 mm × 100 mm, particle size 1.8 µm; Agilent, Santa Clara, CA, USA) for 29 min. The column temperature and flow rate were set to 40°C and 0.4 ml/min, respectively. The mobile phases were 0.1% formic acid in water and acetonitrile. The gradients were as follows: 5% solvent system B for 0 to 1.0 min, 15% solvent system B for 1 to 4 min, 35% solvent system B for 4 to 11 min, 50% solvent system B for 11 to 17 min, 100% solvent system B for 19 to 25 min, and 100% to 0% for 26 to 29 min.

The MS/MS experiments were conducted in negative and positive ion modes with the following parameters: ion voltage of 5.5 kV for positive ion mode and −4.5 kV for negative ion mode, nebulizer gas (gas 1) of nitrogen at 50 psi, heater gas (gas 2) of 50 psi, curtain gas of 20 psi, and turbo spray temperature of 450°C. For a targeted analysis, multiple reaction monitoring (MRM) mode was used, and the optimized conditions for each compound were applied to the mass using flow injection of individual standard compound solutions (100 ng/ml).

### Primary Culture of Human Uterine Leiomyoma Cells

hUtMCs were collected from total 12 patients (average age, 43.83 ± 1.902 years) undergoing curative surgery after obtaining their informed consent. None of them was in menopause and hormonal therapy. Surgically removed uterine leiomyoma was diagnosed as uterine leiomyoma by a pathologist. This study was approved by the ethics committee of Keimyung University School of Medicine (IRB no. 09-156).

The obtained tissue samples from surgery were washed twice in cold phosphate-buffered saline (PBS) before being minced into 5-mm pieces in a sterile culture dish. The minced pieces were transferred into 50-ml conical tubes containing Hank’s balanced salt solution (HBSS; Sigma-Aldrich, St Louis, MO, USA) supplemented with 25 mmol/L 2-[4-(2-hydroxyethyl)piperazin-1-yl] ethanesulfonic acid, 100 U/ml, penicillin 100 U/ml, streptomycin 100 µg/ml, Fungizone^®^ (amphotericin B) 25 ng/ml, 1.5 mg/ml collagenase IV (Sigma-Aldrich), and 0.2 mg/ml of DNase I (Roche Diagnostics, Mannheim, Germany).

All the tubes were incubated at 37°C in a water bath with gentle agitation for 3 h. Undigested tissue was filtered, and the cells were centrifuged at 200*g* for 5 min. The pellet was rinsed once with HBSS and dispersed in complete medium composed of Dulbecco’s modified Eagle’s medium:Nutrient Mixture F-12 (DMEM/F-12, GIBCO, Thermo Fisher Scientific, 11320033) with 10% fetal bovine serum and 100 U/ml antibiotics (GIBCO, Thermo Fisher Scientific, 15240062). Since uterine leiomyoma is a benign and well-demarcated, primary-cultured leiomyoma cells are presumed to be originated from leiomyoma cells. The primary-cultured leiomyoma that showed features of homogenous smooth muscle fibers and confirmed expression of desmin, a marker of smooth cell (**Supplementary Figure S1**). Each batch of cells isolated from each patient was regarded as different sample because of variability of cells. Cells after three to five passages were used and were treated with UPA (10 μmol/L, Sigma-Aldrich) or GBH (0–200 µg/ml).

### Cell Viability Assay

The number of viable cells was determined by the colorimetric 3-(4,5-dimethylthiazol-2-yl)-2,5-diphenyltetrazolium bromide (MTT) assay (Mosmann, 1983). The cells were seeded at a density of 1 × 10^4^ cells/ml in a 96-well plate and then cultured to allow adhesion to the plate. Following this pre-incubation period, the culture medium was replaced with the experimental medium supplemented with GBH.

The normal myometrial cells purchased from ATCC (ACTT, Manassas, VA, USA), and cell viability was evaluated. The culture medium was supplemented with GBH at 0, 10, 30, 50, 100, 300, 600, and 1,000 μg/ml for 24 h to determine the adequate concentration that might not affect normal cells with short-term treatment. Next, we investigated the time-dependent anti-neoplastic effect of GBH to determine the appropriate treatment time under non-toxic concentrations.

After the addition of the MTT reagent, the samples were incubated for an additional 4 h at 37°C. The intensity of the purple color formed is proportional to the number of viable cells. Optical density (OD) was measured at 540 nm. Cell survival was calculated by subtracting the background OD for medium alone, and the values were normalized by dividing the OD of the test wells by the OD of the control (untreated) wells. The data were calculated from triplicate experiments with eight samples per group.

### Morphological Analysis of Apoptotic Cells

Apoptotic bodies were detected, and nuclear morphologies were examined by 4′,6-diamidino-2-phenylindole (DAPI) staining. The experiment was carried out in 6-well microplates with glass cover slides. The hUtMCs (3 × 10^4^/ml) were incubated overnight to allow adhesion to the plate and were treated with UPA or GBH for 48 h. Subsequently, the cells were washed with PBS, fixed in a 4% paraformaldehyde PBS solution for 5 min, and stained with DAPI (final concentration, 300 nmol/L; Thermo Fisher Scientific) for 10 min in the dark. The cells were then examined for nuclear morphology, and images were obtained by fluorescence microscopy.

### Protein Extraction and Western Blot Analysis

UPA-treated or GBH-treated cells were harvested in radioimmunoprecipitation (RIPA) assay buffer (Thermo Fisher Scientific) containing an ethylenediaminetetraacetic acid-free protease inhibitor cocktail (Roche, Mannheim, Germany).

To identify the involvement of GBH effect in caspase pathway/p53 pathway, hUtMCs were pre-incubated with Z-DEVD-FMK (R&D system, Minneapolis, MN, USA), caspase inhibitors or Pifithrin-α (Sigma-Aldrich), p53 inhibitors at 37°C for 30 min, and then were further incubated with GBH (50, 100 μg/ml) at 37°C for 48-h incubation and subjected into Western blot analysis.

Protein concentrations of the cell lysates were determined using a bicinchoninic acid protein assay kit (Bio-Rad, Hercules, CA, USA) following the manufacturer’s protocol. The cell lysates (40 μg) were separated by sodium dodecyl sulfate-polyacrylamide gel electrophoresis using 4% to 20% Mini-PROTEAN^®^ TGX™ Gels (Bio-Rad) and then transferred to nitrocellulose membranes (Millipore, Billerica, MA, USA). The membranes were blocked with Tris-buffered saline containing 5% non-fat milk for 1 h. Next, the membranes were incubated with primary antibodies against Bax (1:500 Santa Cruz Biotechnology, Santa Cruz, CA, USA), Bcl-2 (1:1,000; SC-7382, Santa Cruz Biotechnology), p53 (1:1,000; SC-126, Santa Cruz Biotechnology), caspase-9 (1:1,000, SC-8355, Santa Cruz Biotechnology), caspase-3 (1:1,000; 9661S; Cell Signaling Technology, Beverly, CA, USA), cleaved PARP (1:1,000; 9541; Cell Signaling Technology) and β-actin (1:1,000; SC-47778; Santa Cruz Biotechnology) overnight at 4°C. After reaction with horseradish peroxidase (HRP)-conjugated secondary antibodies (goat anti-rabbit IgG-pAb-HRP conjugate, ADI-SAB-300, and goat anti-mouse IgG F-pAb-HRP conjugate, ADI-SAB-100; Enzo Life Sciences, Farmingdale, NY, USA), bands on the membranes were visualized using an enhanced chemiluminescence system (Thermo Fisher Scientific). The density of each band was analyzed using the ChemiDoc XRS Imaging System (Bio-Rad).

### Detection of Mitochondrial Reactive Oxygen Species Production

To detect superoxide levels in the mitochondria, MitoSOX Red (Molecular Probe, Thermo Fisher Scientific, M36008), MitoTracker Green FM (Molecular Probe, Thermo Fisher Scientific, M7514), and Mitochondrial superoxide detection kit (Abacm, Cambridge, UK) were used according to the manufacturer’s protocol, respectively. Briefly, the cells were incubated with UPA (10 μmol/L) or GBH (10–200 μg/ml) for 48 h and then loaded with MitoSOX Red (5 μmol/L) and MitoTracker Green FM (100 mmol/L) in Hanks’ Balanced Salt Solution (HBSS) (GIBCO, Thermo Fisher Scientific, 14175095) at 36°C for 10 min. After the cells were rinsed with PBS, each slide was covered with ProLong^®^ Gold anti-fade mountant with DAPI (Life Technologies, Thermo Fisher Scientific, P36935).

For mitochondrial superoxide detection, hUtMCs were seeded 3 × 10^4^/ml in a 6-well plate and were treated with UPA (10 μmol/L), GBH (10 – 200 μg/ml), and Mito-TEMPO (50 μmol/L) for 48 h. After treatment, loaded with loaded with MitoROS 580 at 37°C for 1 h. A fluorescent intensity was measured at an excitation wavelength of 540 nm and an emission wavelength of 590 nm using a SpectraMax Gemini XPS/EM fluorescence plate reader (Molecular Devices, LLC., San Jose, CA, USA). And, the fluorescence signal was measured using fluorescence microscope (Olympus, Tokyo, Japan).

### Flow Cytometry

hUtMCs were plated in 6-well plates at a density of 1 × 10^5^ cells/well and were treated with UPA (10 μmol/L), GBH (10–200 μg/ml) for 48 h. The cells were harvested and washed with PBS containing with 1% FBS, followed by staining with annexin V-FITC (50 µl/ml) (Thermo Fisher Scientific) and propidium iodide (PI) (1 µl/ml) (Sigma Aldrich) for 15 min at room temperature. The population of apoptotic cells was analyzed using FACS Calibur (BD Biosciences, San Jose, CA) and the FlowJo software (version 10).

### Statistical Analysis

Data are expressed as the mean ± standard error of mean (SEM). One- and two-way analyses of variance with Tukey’s post-tests were carried out using GraphPad Prism (version 7.0). Values of *P* < 0.05 indicated statistical significance.

## Results

### Identification of the Phytochemical Compounds in GBH

**Figure 1:**
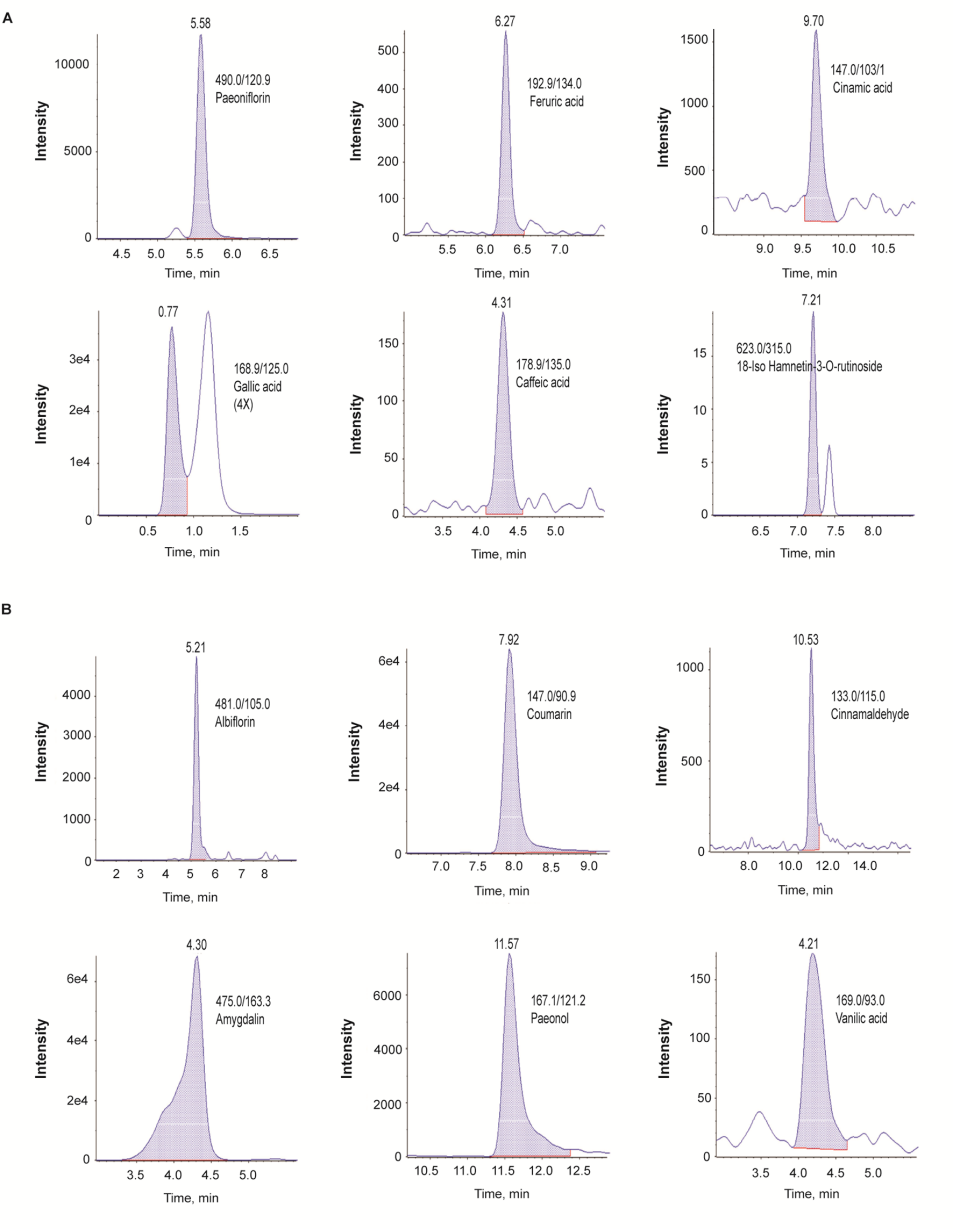
Identification of phytochemicals in GBH by using the multiple reaction monitoring mode of LC-Q-trap mass spectrometry. **(A)** Negative scan modes. **(B)** positive scan modes. GBH, Gyejibongnyeong-hwan.

In the LC-MS/MS negative-ion mode, we identified paeoniflorin, ferulic acid, cinnamic acid, gallic acid, caffeic acid, and 18-iso-rhamnetin-3-O-rutinoside (**Figure 1A**); in the positive mode, we identified albiflorin, coumarin, cinnamaldehyde, amygdalin, paeonol, and vanillic acid (**Figure 1B**).

### Effects of GBH on hUtMC Viability

**Figure 2:**
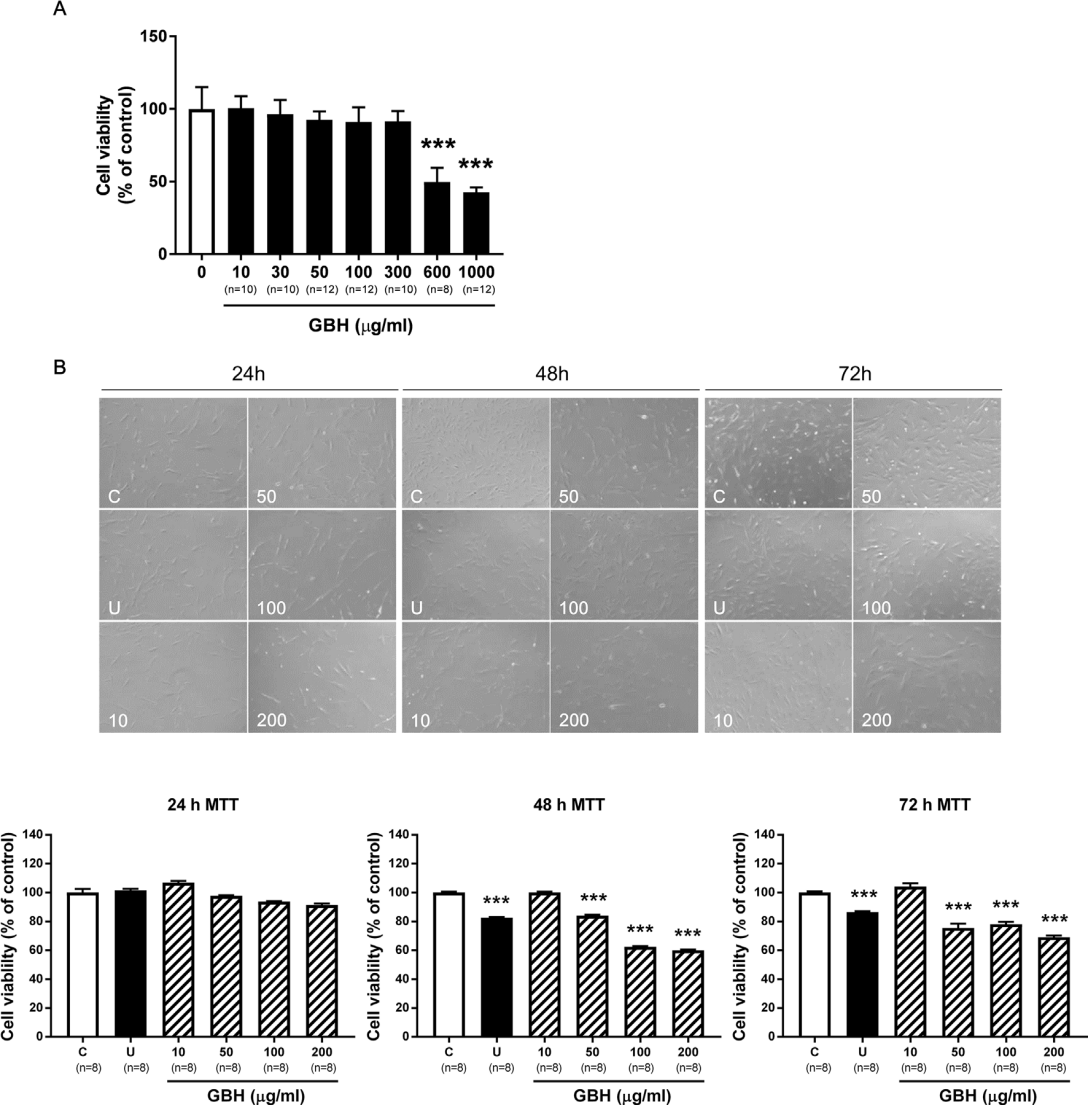
Effects of GBH on hUtMC viability. **(A)** Cell viability evaluated by MTT assay in hUtMCs treated with various concentrations (0–1000 μg/ml) of GBH for 24 h. **(B)** MTT assay after GBH treatment for 24, 48, and 72 h. The upper panel shows images obtained under light microscopy, and the lower panel shows the cell number measurements. Data are expressed as percentages of the basal value (mean ± SEM). ***P < 0.001, vs control. GBH, Gyejibongnyeong-hwan; U, ulipristal acetate; hUtMCs, human uterine myoma cells.

In hUtMCs, 10 to 300 μg/ml of GBH had no cytotoxic effects, but substantially decreased cell viability was observed when the cells were treated with ≥ 600 μg/ml of GBH (*P* < 0.001, vs. control, **Figure 2A**). This indicated that high concentrations of GBH showed cytotoxic effects against hUtMCs. In subsequent experiments, GBH < 300 μg/ml was utilized, which was not cytotoxic. In addition, cell viability of normal myometrial cells decreased from 600 μg/ml of GBH (*P* < 0.001, vs. control, **Supplementary Figure S2**). From result of MTT assay, we estimated IC50 of GBH as 726.1 mg/l.

We next determined the treatment time. Although differences were observed as early as 24 h (**Figure 2B**, upper panel), there were no statistically significant differences in cell number between the control and GBH-treated groups at 24 h. However, after 48 h, cell viability was significantly lower in cells treated with GBH (≥50 μg/ml) than in that the control cells (*P* < 0.001) (**Figure 2B**, lower panel). Cell viability decreased at 72 h but was similar to that at 48 h. These results probably reflected changes in cell proliferation over time, and we used 48 h as the treatment time in subsequent experiments.

### GBH Altered Cell Morphology and Apoptosis Signals in hUtMCs

**Figure 3:**
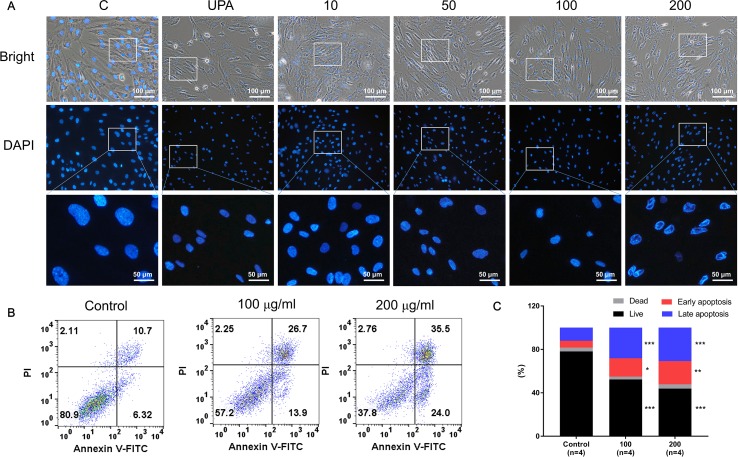
GBH-induced apoptosis of hUtMC. **(A)** Morphology of hUtMCs after treatment with GBH. The outer panel shows an image obtained by light microscopy, and inner panel shows an image obtained by fluorescence microscopy (Magnification 200 ×). **(B)** Apoptosis of hUtMCs was analyzed using flow cytometry and the FlowJo software after staining with propidium iodide (PI) and annexin V-FITC. *P < 0.05, **P < 0.01, ***P < 0.001, vs control.

**Figure 3A** shows the differences in nuclear morphology among nonviable and viable cells and untreated controls after 48 h. The nuclei of cells were round and homogeneously stained in the control group; however, following treatment with over 50 µg/ml of GBH, the cells displayed marked shrinking of nuclei and apoptotic bodies. In addition, 100 and 200 μg/ml of GBH significantly increased the percentage of early (control vs 100, *P* < 0.05; control vs 200, *P* < 0.01) and late (control vs 100, *P* < 0.001; control vs 200, *P* < 0.001) apoptotic cells in dose-dependent manners (**Figures 3B, C**).

**Figure 4:**
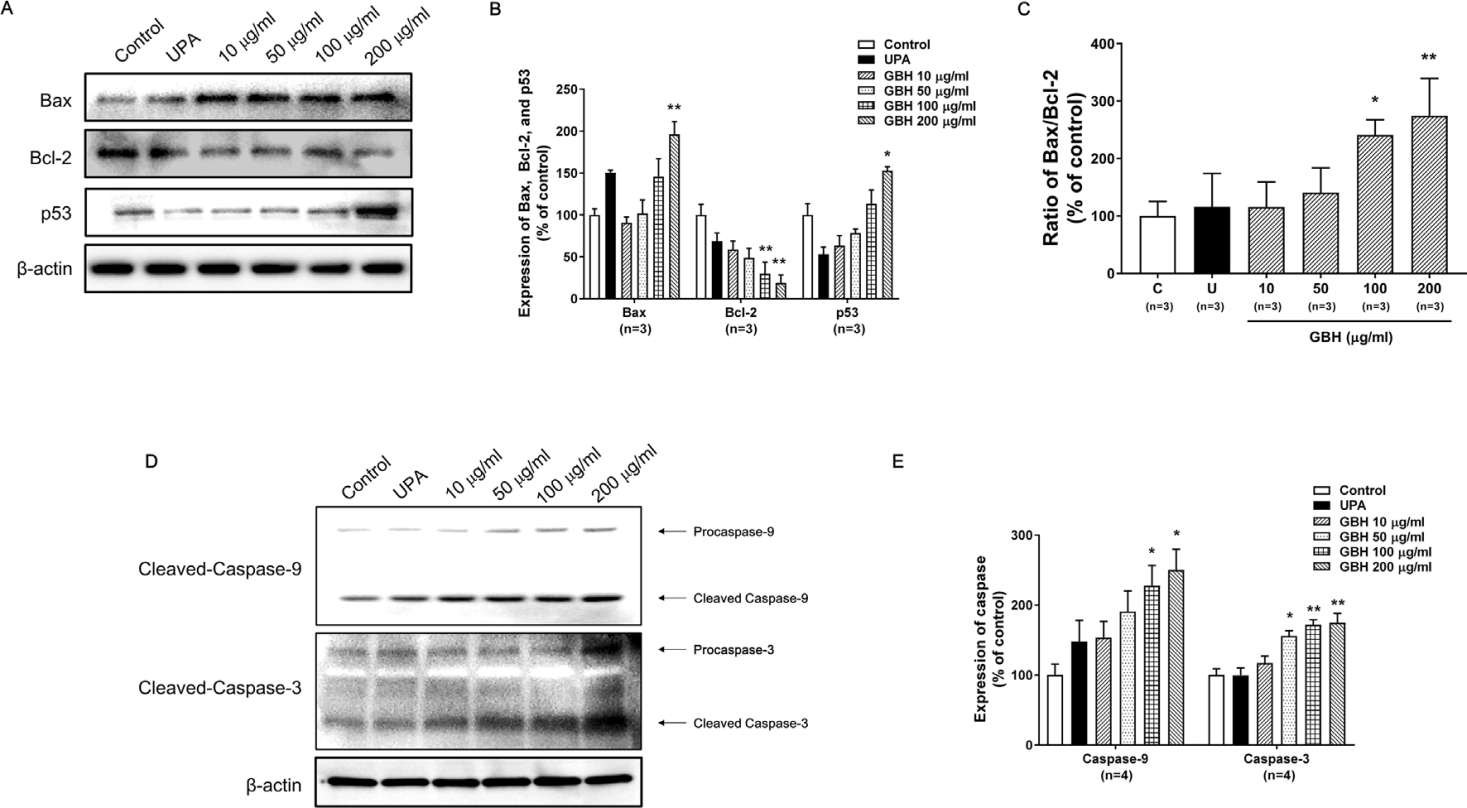
Western blot analyses and the Bax : Bcl-2 ratio in GBH-treated or UPA-treated hUtMCs at 48 h. **(A)** Western blot analyses of Bax, Bcl-2, and p53. **(B)** Quantification of Bax, Bcl-2, and p53 expression. **(C)** The Bcl-2:Bax ratio and protein expression levels. **(D)** Western blot analyses of caspase-3/-9. **(E)** Quantification of expression of caspase-3/-9. The results are presented as means ± SEM of three independent experiments. *P < 0.05, **P < 0.01, vs control. GBH, Gyejibongnyeong-hwan; UPA, ulipristal acetate; hUtMCs, human uterine myoma cells.

The Western blot analysis showed that GBH increased p53 and Bax expression, but decreased Bcl-2 expression in a dose-dependent manner (**Figures 4A, B**). In particular, 100 (*P* < 0.05) and 200 μg/ml (*P* < 0.01) of GBH significantly increased the ratio of Bax to Blc-2 expression (**Figure 4C**). In addition, GBH increased the protein expression of pro and cleaved form of caspase-9 and -3 in a dose-dependent manner (**Figure 4D** and **Supplementary Figure S3** for raw data). In particular, 100 (*P* < 0.05) and 200 μg/ml (*P* < 0.05) of GBH significantly increased expression of caspase 9, and 50 (*P* < 0.05), 100 (*P* < 0.01), and 200 (*P* < 0.01) μg/ml of GBH significantly increased expression of caspase 3 (**Figure 4E**).

**Figure 5:**
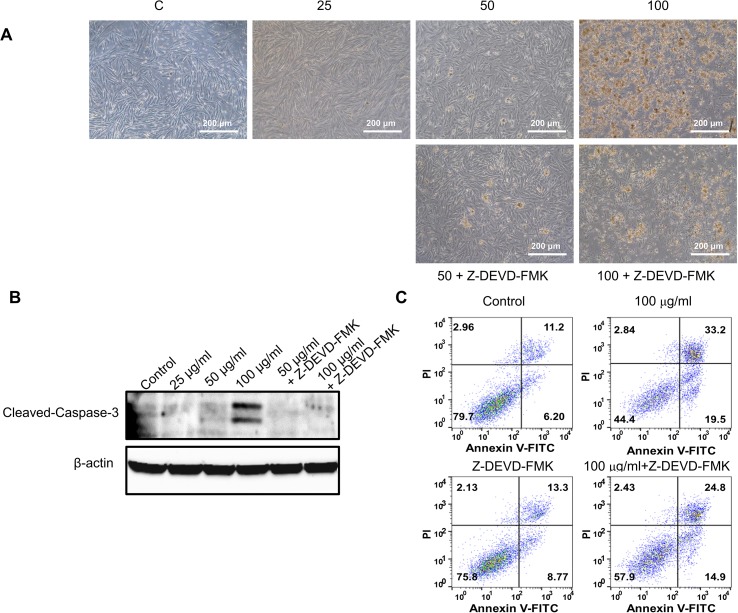
Effect of pan-caspase inhibitor on GBH-induced apoptosis of hUtMC. **(A)** The hUtMC treated with GBH in the presence/or absence of Z-DEVD-FMK, pan-caspase inhibitor **(B)** Western blot analyses of cleaved caspase 3. **(C)** Apoptosis of hUtMCs was analyzed using flow cytometry and the FlowJo software after staining with propidium iodide (PI) and annexin V-FITC.

**Figure 6:**
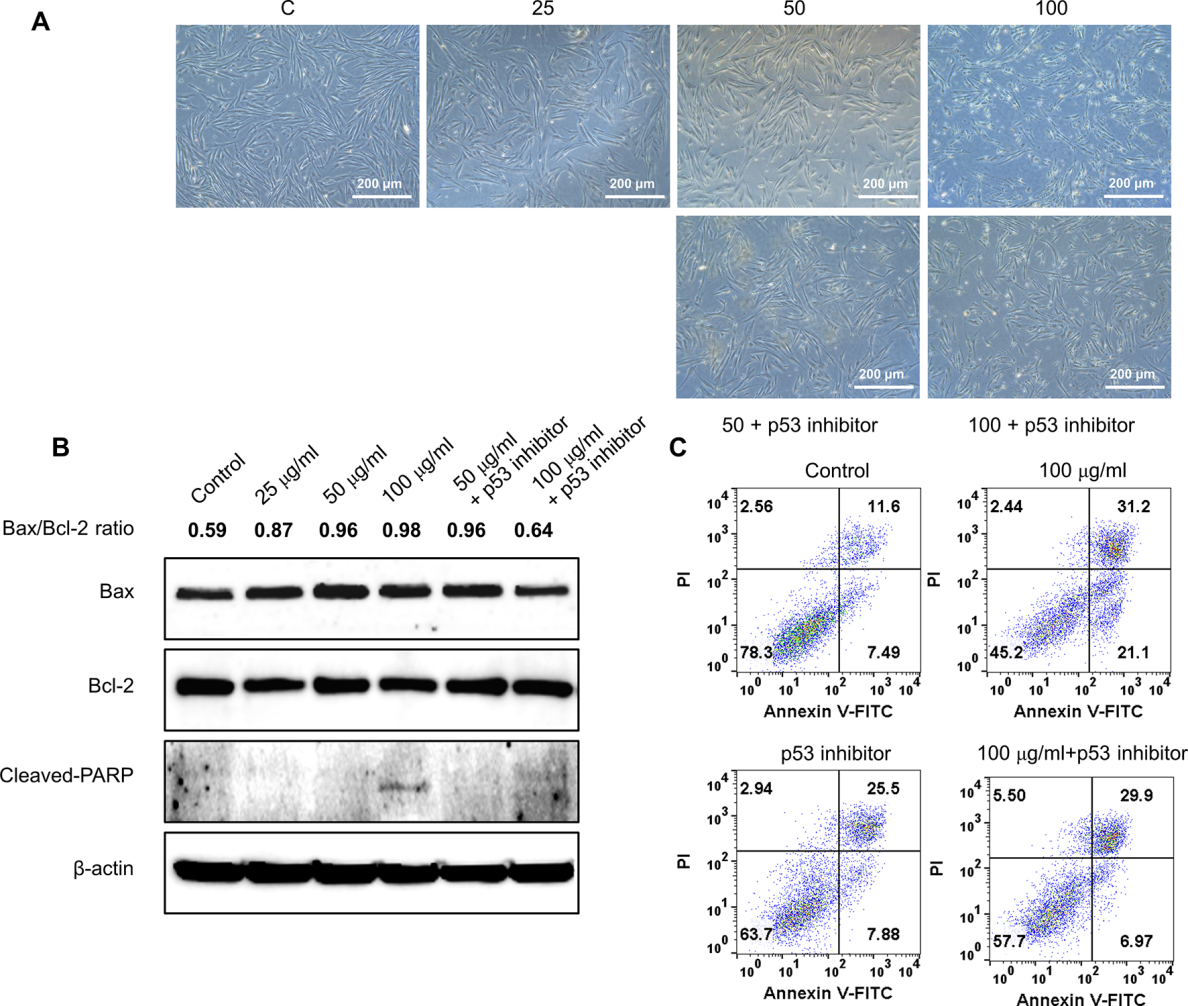
Effect of p53 inhibitor on GBH-induced apoptosis of hUtMC. **(A)**: The hUtMC treated with GBH in the presence/or absence of, p53 inhibitor. **(B)**: Western blot analyses of Bax, Bcl-2, and cleaved PARP. **(C)**: Apoptosis of hUtMCs were analyzed using flow cytometry and the FlowJo software after staining with propidium iodide (PI) and annexin V-FITC.

To further validate the effect of GBH on apoptosis, we executed preliminary tests using a pan-caspase inhibitor and p53 inhibitor. The treatment of 100 μg/ml of GBH induced apoptosis (**Figure 5A**), whereas 100 μg/ml of GBH with Z-DEVD-FMK, a pan-caspase inhibitor, reduced expression of GBH-mediated cleaved caspase-3 (**Figure 5B**) and the percentage of apoptosis cell (**Figure 5C**), which indicated caspase-dependent apoptotic effect of GBH. In addition, 100 μg/ml of GBH with pifithrin-α, p53 inhibitor, also slightly rescued 100 μg/ml of GBH-mediated apoptotic effect (**Figure 6A**), resulting reduced expression of Bax and Cleaved PARP (**Figure 6B**) and the percentage of apoptosis cell (**Figure 6C**). These indicated that p53 may not be main pathway but involved in GBH-mediated apoptotic effect.

### GBH Induced Apoptosis *via* Mitochondrial Oxidative Stress in hUtMCs

**Figure 7:**
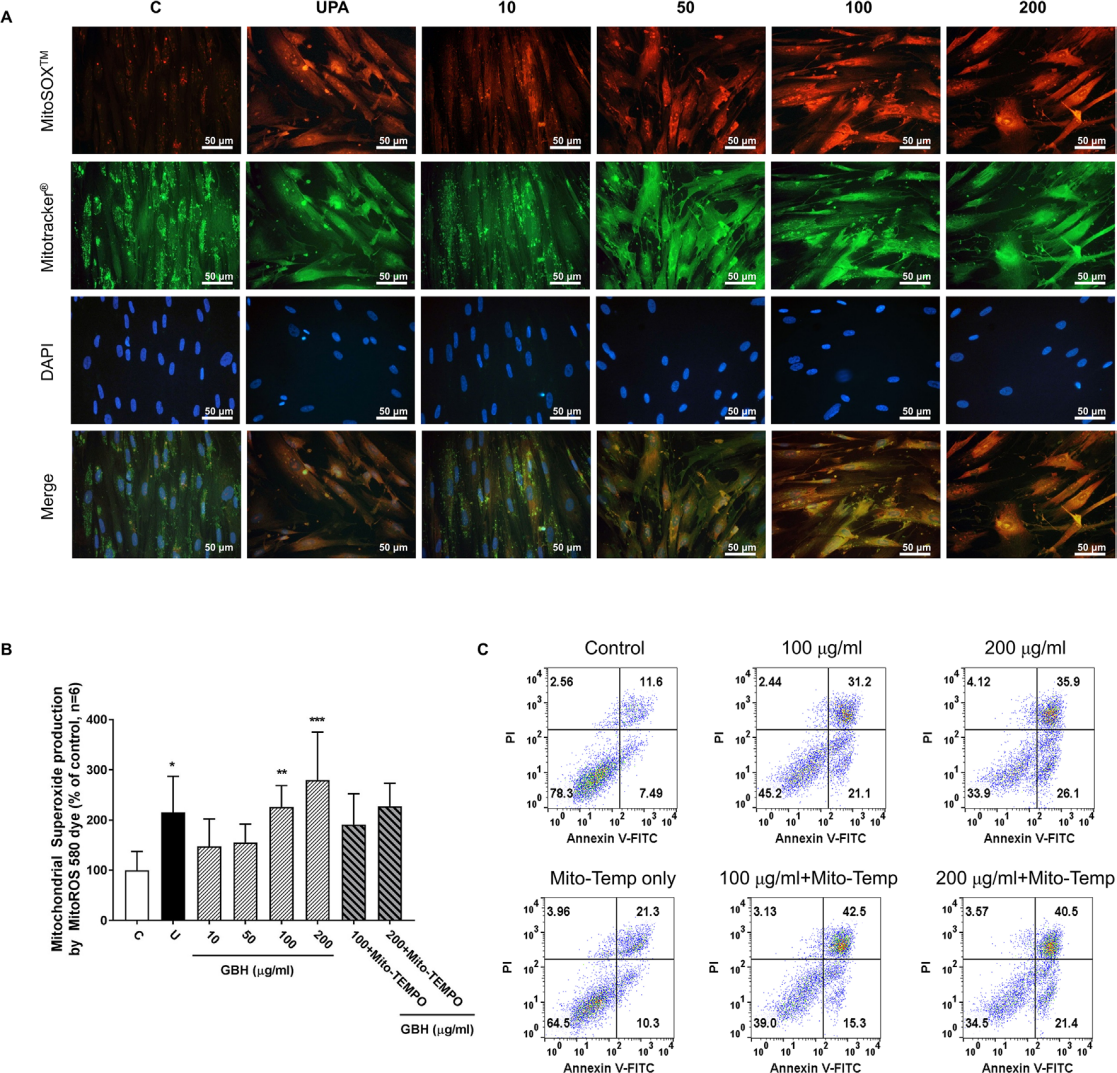
Change of mitochondrial reactive oxygen species (ROS) generation in hUtMC cells after treatment with GBH for 48 h. **(A)**: MitoSOX Red, Mitotracker Green, and DAPI staining images obtained from hUtMC cells treated with GBH. **(B)**: Mitochondrial superoxide (red) were detected by fluorescence 540/590 nm after staining the MitoROS 580 dye. **(C)**: Apoptosis of hUtMCs treated with GBH in the presence/or absence of MitoTEMP, a mitochondria ROS scavenger, were analyzed using flow cytometry and the FlowJo software after staining with propidium iodide (PI) and annexin V-FITC. * *P* < 0.05, ** *P* < 0.01, *** *P* < 0.001, vs control. GBH, Gyejibongnyeong-hwan; UPA, ulipristal acetate; hUtMCs, human uterine myoma cells; MitoROS, mitochondrial superoxide.

Since reactive oxygen species (ROS) play a critical role in the induction of apoptosis (Vakifahmetoglu-Norberg et al., 2017), we investigated whether the GBH induces mitochondrial ROS production in hUtMCs. The results showed that GBH enhanced MitoSOX red signals, mitochondrial superoxide indicator images, in a dose-dependent manner (**Figure 7A**). In merged images of DAPI, MitoSOX, and Mitotracker signals, GBH-treated apoptotic hUtMCs showed enhanced red signals within the mitochondria, which was similar to the observations in UPA-treated hUtMCs.

In addition, GBH significantly increased mitochondrial ROS in dose-dependent manners (control vs 100, *P* < 0.01; control vs 200, *P* < 0.001), and GBH with MitoTEMPO, a mitochondria ROS scavengers, slightly reduced the mitochondria ROS of hUtMCs (**Figure 7B**). In accordance with this result, preliminary test using GBH with MitoTEMPO showed that the percentage of early apoptotic cells also slightly reduced compared to those of hUtMCs cells treated GBH without MitoTEMPO (**Figure 7C**).

## Discussion

Uterine leiomyoma is not a malignant disease, but it is also the most common benign smooth muscle tumor in reproductive-aged women (Laganà et al., 2017). Nevertheless, treatment strategies for uterine leiomyoma are still limited, and preventive therapies have not been developed. In this study, we evaluated the effect of GBH, a traditional Korean and Chinese medicine, on uterine leiomyomas, and investigated the molecular mechanism underlying anti-neoplastic effect of GBH.

GBH has been used extensively for the treatment of gynecological diseases in Korean medicine. A clinical study reported that GBH significantly improves uterine and myoma volumes and decreases abnormally high hormone levels (Chen et al., 2014). Yi et al. (Yi et al., 2002) also showed that GBH inhibits the proliferation of uterine leiomyoma cells. In the present study, we observed that GBH reduced cell numbers, decreased cell viability, and enabled the formation of apoptotic bodies, accompanied by changes in molecules related to cell death and apoptosis, which were similar to the effects of UPA, the only drug approved for uterine leiomyomas thus far.

As shown in **Figure 4**, GBH regulates p53, which is a central node that integrates several stress response pathways and governs apoptosis, cell cycle arrest, senescence, and other physiological processes (Vousden and Prives, 2009; Speidel, 2010). Specifically, the tumor suppressor activity of p53 is due in part to its ability to initiate the intrinsic mitochondria-mediated pathway for apoptosis by controlling members of the Bcl-2 protein family and by activating the pro-apoptotic member Bax. Given the opposite roles of Bcl-2 and Bax in mitochondrial outer membrane permeabilization, the ratio of Bax to Bcl-2 is widely used as an indicator of apoptosis (Oltvai et al., 1993). In this study, the Bax and Bcl-2 proteins in hUtMCs were up- and down-regulated, respectively, by GBH in a dose-dependent manner, along with up-regulation of p53 expression. Furthermore, GBH significantly altered the ratio of Bax to Bcl2 in a dose-dependent manner (**Figure 4C**). These results show that GBH initiated p53-dependent intrinsic mitochondria-mediated apoptosis.

Additionally, the p53-dependent intrinsic mitochondria-mediated apoptosis pathway induces the release of cytochrome c and thereby triggers caspase-dependent apoptotic cell death (Gogada et al., 2011). Cytochrome c associates with procaspase 9 to form the apoptosome, a protein complex that, through activation of caspase 9, ultimately activates caspase 3 as a final signal of apoptosis (Oltvai et al., 1993; Rudel, 1999; Yang et al., 2002; Salakou et al., 2007; Speidel, 2010; Gogada et al., 2011). In the present study, GBH increased the expression of procaspase-9 and cleaved-caspase-9 and up-regulated cleaved-caspase-3 in a dose-dependent manner, inducing apoptosis by activating caspase fragmentation in GBH-treated hUtMCs. In addition, we confirmed that hUtMCs treated p53 inhibitor with GBH, and pan-caspase inhibitor with GBH rescued GBH-mediated apoptotic effect (**Figures 5** and **6**), which demonstrated that GBH-induced apoptosis is associated with p53 and caspase-dependent apoptotic pathway.

In addition, Rizzello et al. (Rizzello et al., 2017) have reported that uterine myoma are associated with the over-expression of estrogen and progesterone receptors, and a few studies have also reported that GBH reduces estrogen and progesterone levels (Li et al., 2003; Ying, 2012). Thus, apoptosis induced by GHB might be related to estrogen or progesterone signaling, in turn regulating several apoptotic proteins (Lewis-Wambi and Jordan, 2009).

In this study, we observed that GBH exerted anti-neoplastic effects on hUtMCs by inducing apoptosis. Previous studies using antioxidants have reported that ROS act upstream of mitochondrial membrane depolarization, Bax relocalization, and cytochrome c release, executing caspase activation and nuclear fragmentation (Wang et al., 1999; Circu and Aw, 2010; Brentnall et al., 2013). Specifically, mitochondria play crucial roles in ROS production. Thus, we investigated mitochondrial ROS status and observed that GBH inducted ROS in hUtMCs.

Among the major compounds of GBH, several phenolic compounds, such as cinnamic acid, vanillic acid, ferulic acid, caffeic acid, gallic acid, and quercetin, which are known to exert multiple therapeutic effects, were identified. Previous reports have elucidated the effects of phenolic compounds on intracellular redox state; these results support both the anti- and pro-oxidant activities of these compounds, depending on the concentration of the compounds and the cell type (de la Lastra and Villegas, 2007; Heiss et al., 2007). Islam et al. also recently suggested strawberry-induced apoptosis and ROS in hUtMCs due to the combinatorial effect of phenolics and anthocyanins (Islam et al., 2017). Here, we found that three phenolic compounds, cinnamic acid, vanillic acid, and ferulic acid, showed anti-neoplastic effect in hUtMCs (**Supplementary Figure 4 **). In addition, GBH increased mitochondrial superoxide anions as a mechanism of apoptotic induction, and mitochondria ROS scavenger rescued GBH-induced early apoptosis, given that ROS plays an important role in signal transduction leading to apoptosis of hUtMCs.

Our results suggest that GBH increases mitochondrial dysfunction and induces mitochondria-mediated cell death by increasing the Bax/Bcl-2 ratio and ROS levels. The mechanism of action GBH may be similar to that of UPA, which inhibits the proliferation of leiomyoma cells and induces apoptosis by increasing cleaved-caspase-3 expression and decreasing Bcl-2 expression (Maruo et al., 2000). However, GBH increased not only the percentage of cells at early apoptosis but also that of cells at late apoptosis/necrosis in this study. In addition, analysis of apoptotic cell after treatment of GBH with pan-caspase inhibitor/p53 inhibitor/mitochondria ROS scavenger is preliminary data. Thus, further validation tests are needed to verify the effect of GBH on uterine leiomyomas and normal cells.

## Conclusion

Herein, we found that GBH induced early and late apoptosis and inhibited the proliferation of leiomyoma cells. However, further *in vivo* and long-term experiments are needed before GBH can be applied in the clinical setting. In addition, more studies are needed to determine the bioactive compounds and their underlying molecular mechanisms in exerting the GBH effects on uterine fibroids to overcome the limitation of natural crude extracts and to develop GBH as a new drug. Furthermore, the effect of GBH on normal and leiomyoma cells should be compared with avoid any unwanted effects in normal cells. Notwithstanding, the findings of the present study highlight the potential benefits of GBH for the management of uterine leiomyomas.

## Ethics Statement

Human uterine myoma cells (hUtMCs) were collected from patients undergoing curative surgery after obtaining their informed consent. This study was approved by the ethics committee of Keimyung University School of Medicine (IRB No. 09-156).

## Author Contributions

Conceptualization: SL, JJ, EH, and SS. Data curation: SL, EC, and KJ. Funding acquisition: JJ. Investigation: SL. Methodology: SL, JJ, EH, and SS. Project administration: JJ and EH. Resources: SS. Supervision: JJ and EH. Validation: JJ. Visualization: SL. Writing—original draft: SL, JJ, and EH.

## Funding

This study was supported by the Korea Institure of Oriental Medicine (KSN1812190 and KSN1812210) and the National Research Foundation of Korea (NRF) funded by the Ministry of Science and ICT (NRF-2015R1A2A2A01007167).

## Conflict of Interest Statement

The authors declare that the research was conducted in the absence of any commercial or financial relationships that could be construed as a potential conflict of interest.
