# Supplementary material for: Gyejibongnyeong-Hwan (Gui Zhi Fu Ling Wan) Ameliorates Human Uterine Myomas via Apoptosis
Source: Front Pharmacol. 2019 Sep 25;10:1105. doi: 10.3389/fphar.2019.01105 (PMC6774280; doi:10.3389/fphar.2019.01105)
Supplement: Supplementary file 1 [file DataSheet_1.docx]

**Supplementary**

**Gyejibongnyeong-hwan (Gui Zhi Fu Ling Wan) ameliorates human uterine myomas via apoptosis**

**[Suggestion for running title: GBH treatment for uterine myomas**

**So Min Lee,^1^ Eun Som Choi^2,3^ Eunyoung Ha,^3^ So Jin Shin,^2*^ and Jeeyoun Jung^1*^**

^1^Clinical Medicine Division, Korea Institute of Oriental Medicine 1672, Yuseong-daero, Yuseong-gu, Daejeon, 354054, Korea

^2^Department of Obstetrics and Gynecology, Institute for Cancer Research, Keimyung University School of Medicine, Jung-gu, Daegu 41931, Korea

^3^Department of Biochemistry, Institute for Cancer Research, Keimyung University School of Medicine, Jung-gu, Daegu 41931, Korea

^4^Department of Obstetrics and Gynecology, Institute for Cancer Research, Keimyung University School of Medicine, Jung-gu, Daegu 41931, Korea

**Keywords:** Uterine leiomyoma cell; Gyejibongnyeong-hwan; Apoptosis; Bax; Bcl-2

*Correspondence should be addressed to:

Jeeyoun Jung, O.M.D., Clinical Medicine Division, Korea Institute of Oriental Medicine 1672, Yuseong-daero, Yuseong-gu, Daejeon, 354054, Republic of Korea. Tel: +82-42-868-9272, Fax: +82-42-868-9299, E-mail: [jjy0918@kiom.re.kr](mailto:jjy0918@kiom.re.kr)

So Jin Shin, M.D., Department of Obstetrics and Gynecology, Keimyung University School of Medicine, 1095 Dalgubeol-daero, Dalseo-gu, Daegu 42601, Republic of Korea. Tel: +82-53-258-4847, Fax: +82-53-258-4847, E-mail: [hope2014@dsmc.or.kr](mailto:hope2014@dsmc.or.kr)

**Supplementary Methods**

***Cell viability assay***

The Cell Counting Kit-8 (CCK-8, Dojindo, Sunnyvale, CA, USA) assay was used as a qualitative index of cell viability. The CCK-8 assay was used to measure cytotoxicity under starved conditions, which were based on the conversion of a water-soluble tetrazolium salt, 2-(2-methoxy-4-nitrophenyl)-3-(4-nitrophenyl)-5-(2,4-disulfophenyl)-2H-tetrazolium, monosodium salt (WST-8), to a water-soluble formazan dye upon reduction by dehydrogenases in the presence of an electron carrier (1,2). Primay uterine smooth muscle cells (ATCC, Manassas, VA, USA, PCS-460-011) were plated in 96-well plates at a density of 1 × 10^4^ cells per well and then cultured to allow adhesion to the plate. Following this pre-incubation period, the culture medium was replaced with the experimental medium supplemented with GBH at 0, 10, 30, 50, 100, 300, 600, and 1000 μg/mL for 24 h. The live cell count was obtained using CCK-8 according to the manufacturer's protocol. In brief, 10 µL of CCK-8 solution was added to each well, and the samples were incubated for four hours before the absorbance was measured at 450 nm.

***Protein extraction and Western blot analysis***

UPA, GBH, or/and Z-DEVD-FMK-treated cells were harvested in radioimmunoprecipitation (RIPA) assay buffer (Thermo Scientific, Rockford, IL, USA) containing an ethylenediaminetetraacetic acid-free protease inhibitor cocktail (Roche, Mannheim, Germany). Protein concentrations of the cell lysates were determined using a bicinchoninic acid protein assay kit (Bio-Rad, Hercules, CA, USA) following the manufacturer’s protocol. The cell lysates (35 μg) were separated by sodium dodecyl sulfate-polyacrylamide gel electrophoresis using 4–20% Mini-PROTEAN^®^ TGX^TM^ Gels (Bio-Rad) and then transferred to nitrocellulose membranes (Millipore, Billerica, MA, USA). The membranes were blocked with Tris-buffered saline containing 5% non-fat milk for 1 hour. Next, the membranes were incubated with primary antibodies against caspase-3 (1:1000; #9662S, Cell Signaling Technology, Beverly, CA, USA), desmin (1:1000; SC-23879, Santa Cruz) and β-actin (1:1000; SC-47778, Santa Cruz) overnight at 4°C. After reaction with horseradish peroxidase (HRP)-conjugated secondary antibodies (goat anti-rabbit IgG-pAb-HRP conjugate, ADI-SAB-300 and goat anti-mouse IgG F-pAb-HRP conjugate, ADI-SAB-100; Enzo Life Sciences, Farmingdale, NY, USA), bands on the membranes were visualized using an enhanced chemiluminescence system (Thermo Scientific). The density of each band was analyzed using the ChemiDoc XRS Imaging System (Bio-Rad).

***Detection of mitochondrial superoxide***

To assess mitochondrial superoxide detection kit (Abacm, Cambridge, UK) was used according to the manufacturers’ instructions. Briefly, hUtMCs were seeded 3 × 10^4^/mL in a 6-well plate and were treated with UPA (10 μmol/L), GBH (10–200 μg/mL), or MitoTEMPO (50 μmol/L) for 48 h. After treatment, loaded with loaded with MitoROS 580 at 37°C for 1 hour. A fluorescent intensity was measured at an excitation wavelength of 540 nm and an emission wavelength of 590 nm using a SpectraMax Gemini XPS/EM fluorescence plate reader (Molecular devices, LLC., San Jose, CA, USA). And, the fluorescence signal was measured using fluorescence microscope (Olympus, Tokyo, Japan).

**Supplementary Results**

**
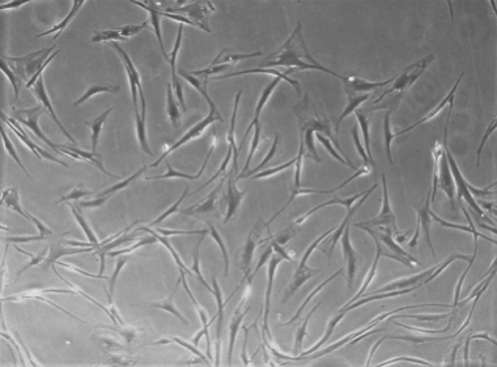

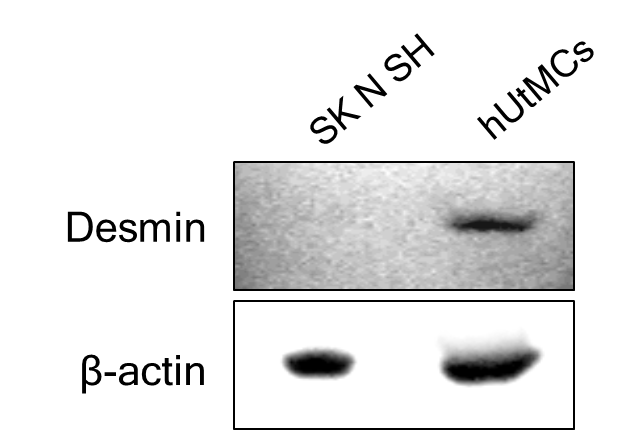
**

**Figure S1.** Primary myometrial cells from women with myoma uterine. The primary-cultured leiomyoma showed that the homogenous smooth muscle fibers and smooth muscle cells markers.


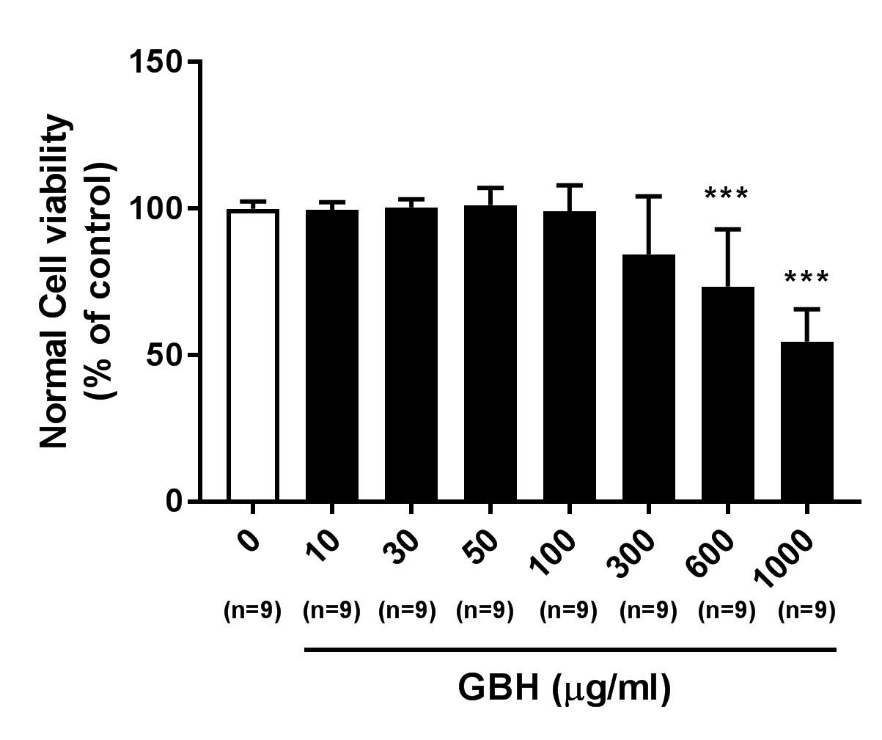


**Figure S2.** Effects of GBH on the normal myometrial cell viability. The normal myometrial cells were treated with various concentrations (0–1000 μg/mL) of GBH for 24 h. Data are expressed as percentages of the basal value (mean ± SEM of independent experiments with three plates). ***P < 0.001, vs the control. GBH: Gyejibongnyeong-hwan; SEM: standard error of the mean.

**
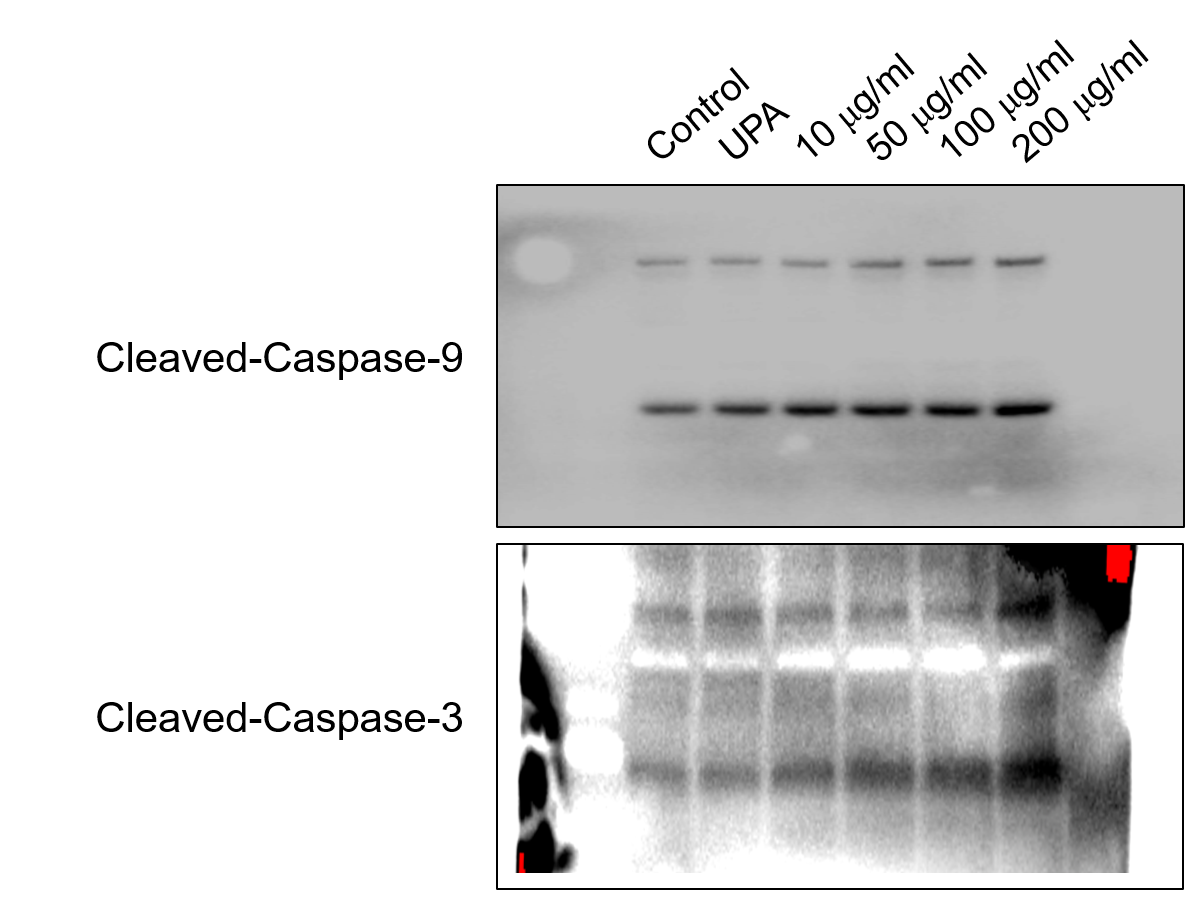
 Figure S3.** Raw data of western blot analysis of cleaved-caspase-9 and cleaved-caspase-3 expression in hUtMC.


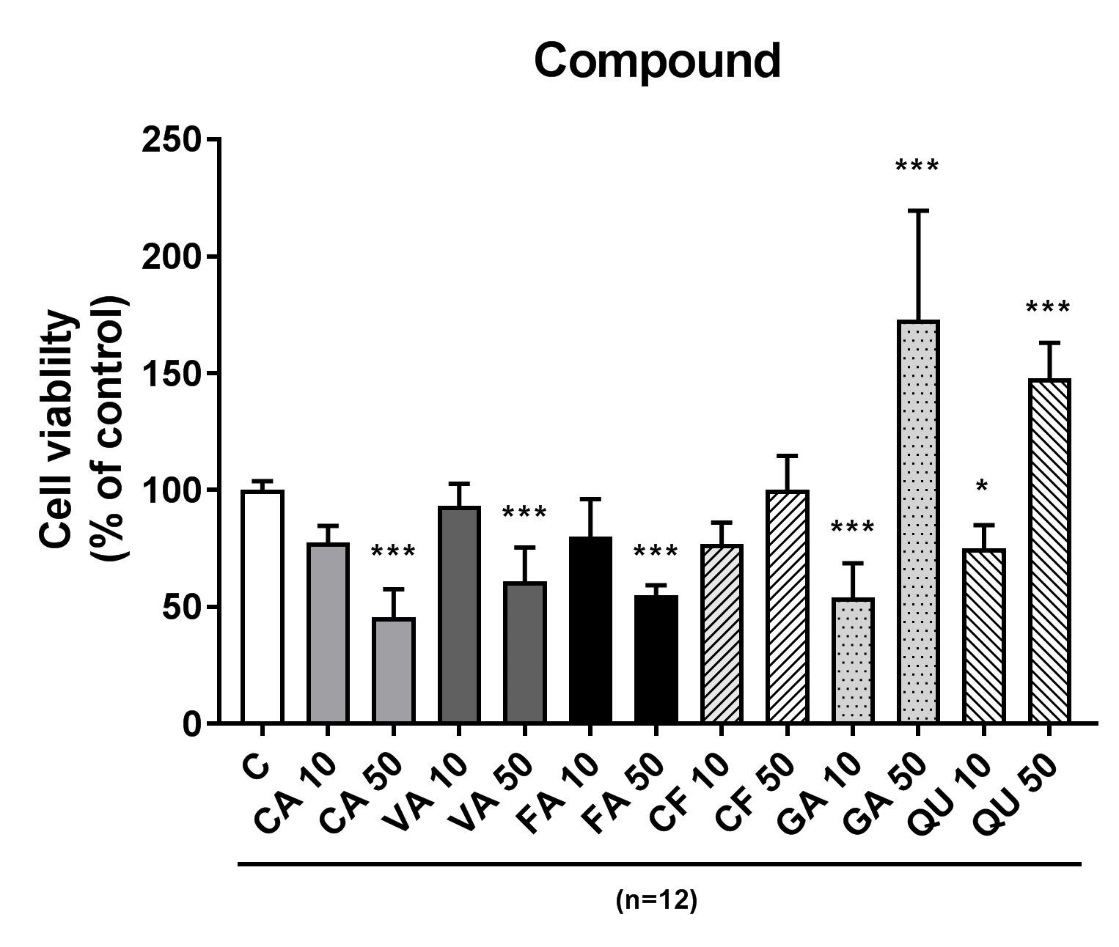


**Figure S4.** Effects of GBH six compound on hUtMC viability. The hUtMCs were treated with two concentrations (10–50 μmol/L) of GBH six compound for 24 h. Data are expressed as the mean ± SEM of duplicate measurements. * *P* < 0.05, ** *P* < 0.01, *** *P* < 0.01 vs the control. C: control; CA: cinnamic acid; VA: vanillic acid; FA: ferulic acid; CF: caffeic acid; GA: gallic acid; QU: quercetin; hUtMCs: human uterine myoma cells; SEM: standard error of the mean.

**Supplementary Reference**

1. Han SB, Shin YJ, Hyon JY, Wee WR. Cytotoxicity of vorico nazole on cultured human corneal endothelial cells. Antimicrob Agents Chemother (2011) 55:4519-4523.

[PubMed] https://www.ncbi.nlm.nih.gov/pubmed/21768517

2. Ishiyama M, Tominaga H, Shiga M, Sasamoto K, Ohkura Y, Ueno K. A combined assay of cell viability and in vitro cytotoxicity with a highly water-soluble tetrazolium salt, neutral red and crystal violet. Biol Pharm Bull (1996) 19:1518-1520.

[PubMed] https://www.ncbi.nlm.nih.gov/pubmed/8951178
